# Moving on with allergy: transition in allergy services

**DOI:** 10.1186/2045-7022-5-S3-P6

**Published:** 2015-03-30

**Authors:** Anna Calvert, Nicola Jay

**Affiliations:** 1 Sheffield Children's Hospital NHS Trust, Sheffield, UK

## Context

Food Allergy is common in the paediatric population and increasingly persists into adulthood. Allergies have a significant impact on quality of life and adolescents are over represented in significant and fatal reactions. Associated asthma increases the risk of a fatal reaction. Provision of allergy services within adult medicine can be limited and there is often no clear structure for transitioning patients from paediatric to adult practice leaving a high risk population with inadequate provision.

## Aims

In order to develop a transition pathway, we looked at all the young people approaching the age of 16 in one of our allergy clinics over a twelve month period.

## Objectives

To assess the number of young people approaching sixteen and define the characteristics of this group to develop an understanding of what further medical input they would require.

## Results

There were 74 young people identified from one clinic in a twelve month period who were awaiting transition. Food allergy was the commonest allergy encountered (43 peanut, 27 tree nut, 2 seafood, 6 fish, 5 sesame seed, 17 other) with 27 of the young people having two or more allergies. Anadrenaline autoinjector was part of the management plan in 52 individuals. Atopic co-morbidities were present in 52 patients, with asthma being the most prevalent. In 25 patients 2 or more atopic conditions were present.

## Conclusions

We have shown that food allergy continues to be a problem for young people approaching adulthood and that this cohort of patients requires adrenaline auto injectors and have significant comorbidities. This indicates that these patients are at risk from significant allergic reactions and need a clear pathway for their continued management. Food allergy is no longer just a problem for paediatricians and strenuous efforts are required to provide services for adolescents, which are accessible and appropriate for their needs.

